# Supplementary material for: A New Census of Protein Tandem Repeats and Their Relationship with Intrinsic Disorder
Source: Genes (Basel). 2020 Apr 9;11(4):407. doi: 10.3390/genes11040407 (PMC7230257; doi:10.3390/genes11040407)
Supplement: Supplementary file 1 [file genes-11-00407-s001.pdf]

genes

MDPI

Supplementary Materials

A New Census of Protein Tandem Repeats and their Relationship with Intrinsic Disorder

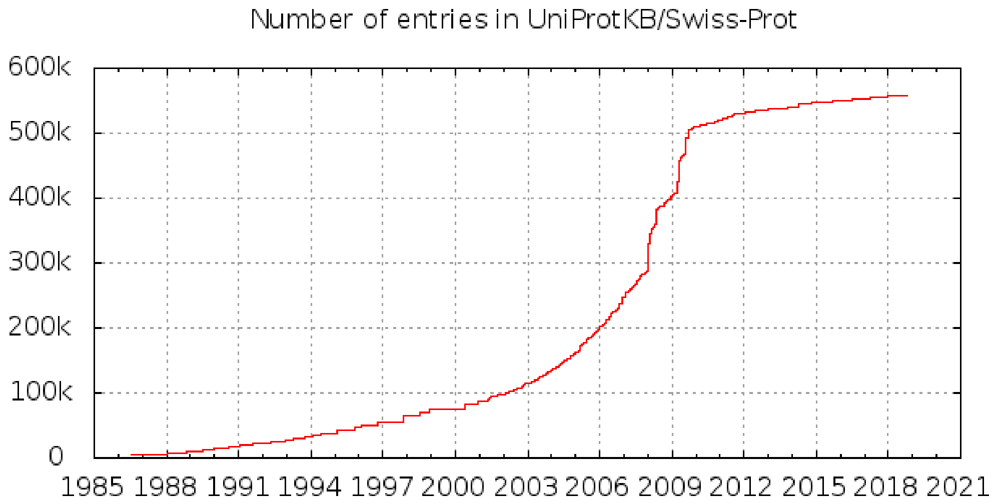

Figure S1. Summary of the growth of UniProtKB/Swiss-Prot protein knowledgebase. The last protein census dates back to the year 1999 [1]. Since then, the entries in the UniProtKB/Swiss-Prot protein knowledgebase are grown more than seven-fold. Figure from release 2018_09 statistics. https://web.expasy.org/docs/relnotes/relstat.html, retrieved on 2018/10/17.

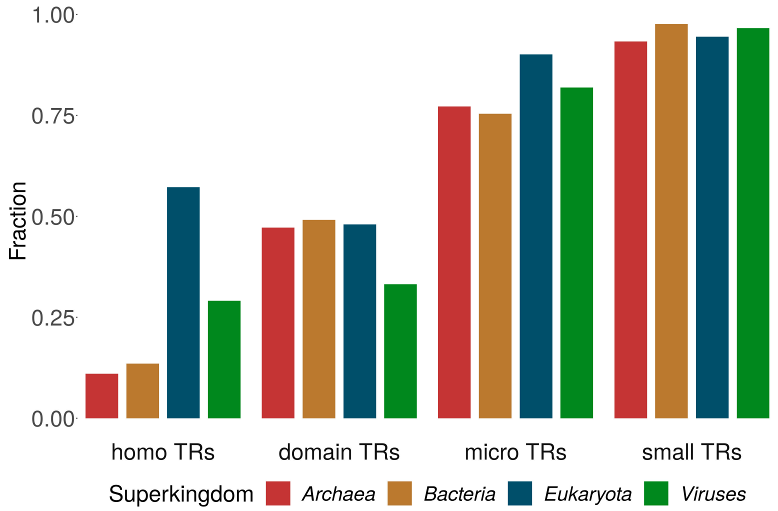

Figure S2. Proteins with ≥ 4 distinct TR regions are sorted by their TR type and shown in different colours for each super kingdom. One can clearly see, that overall super kingdoms small TRs dominate in proteins with many distinct TR regions.

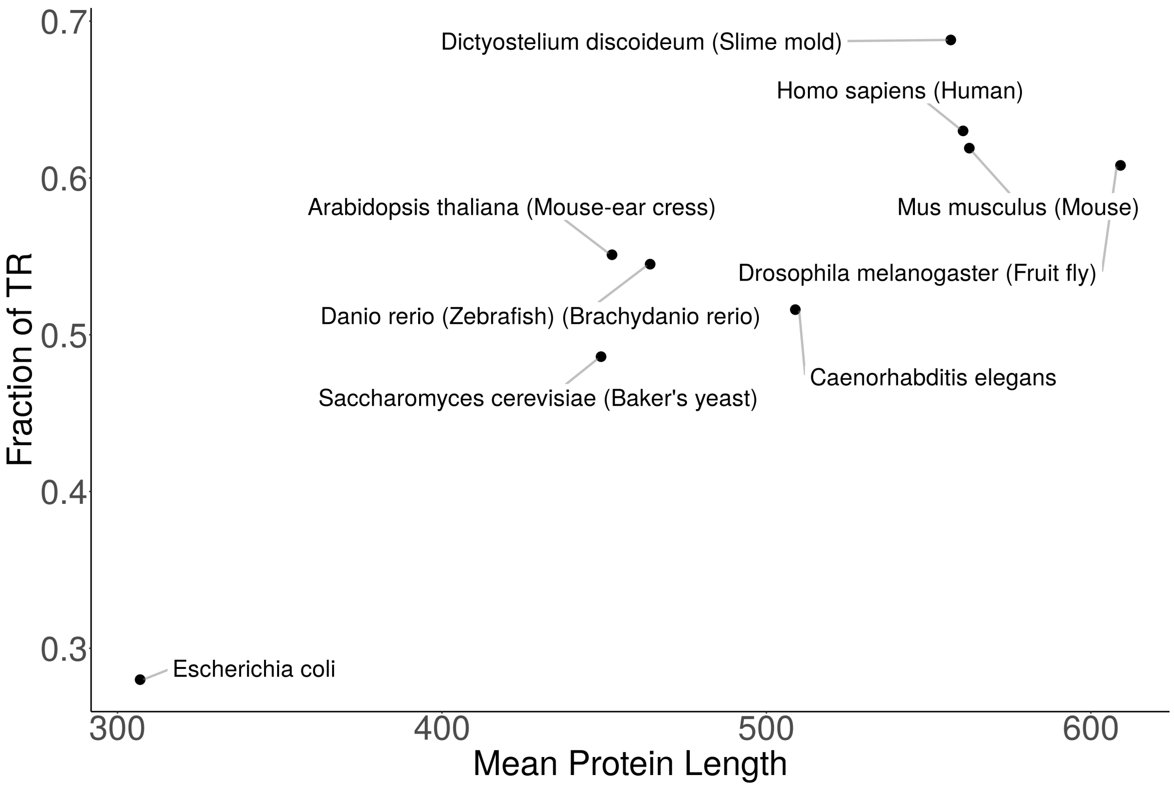

Figure S3. The fraction of proteins containing TRs overall protein entries in UniProtKB/Swiss-Prot is shown for a selection of heavily investigated species and displayed as function of the mean protein length. The tendency of more complex organisms having more and longer TRs can be seen.

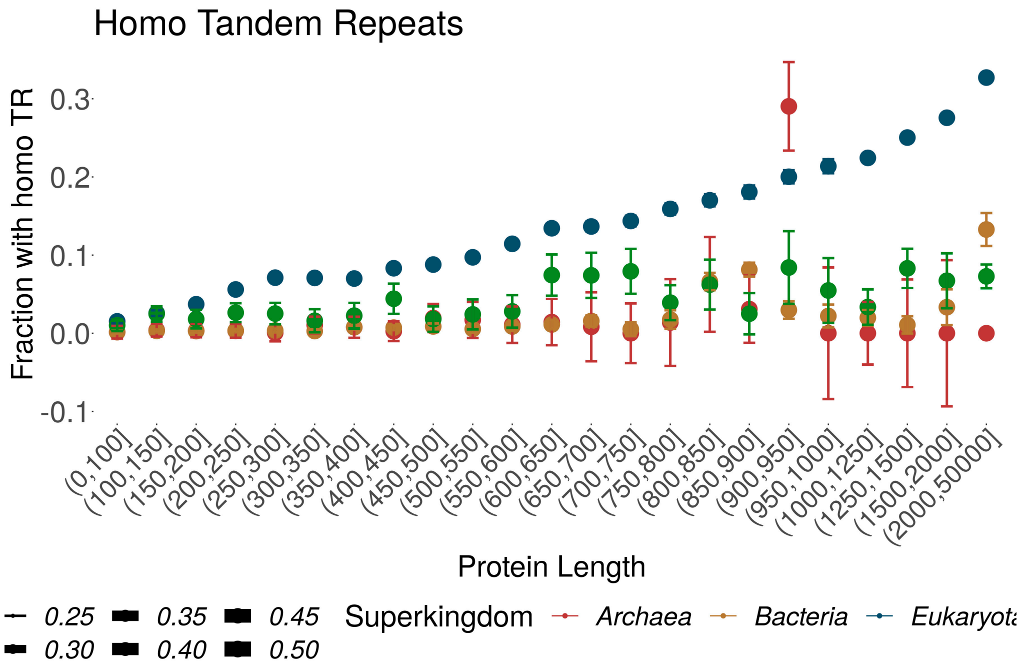

Figure S4. The fraction of proteins with homo TRs as a function of sequence length by kingdom resulting in a linear relationship.

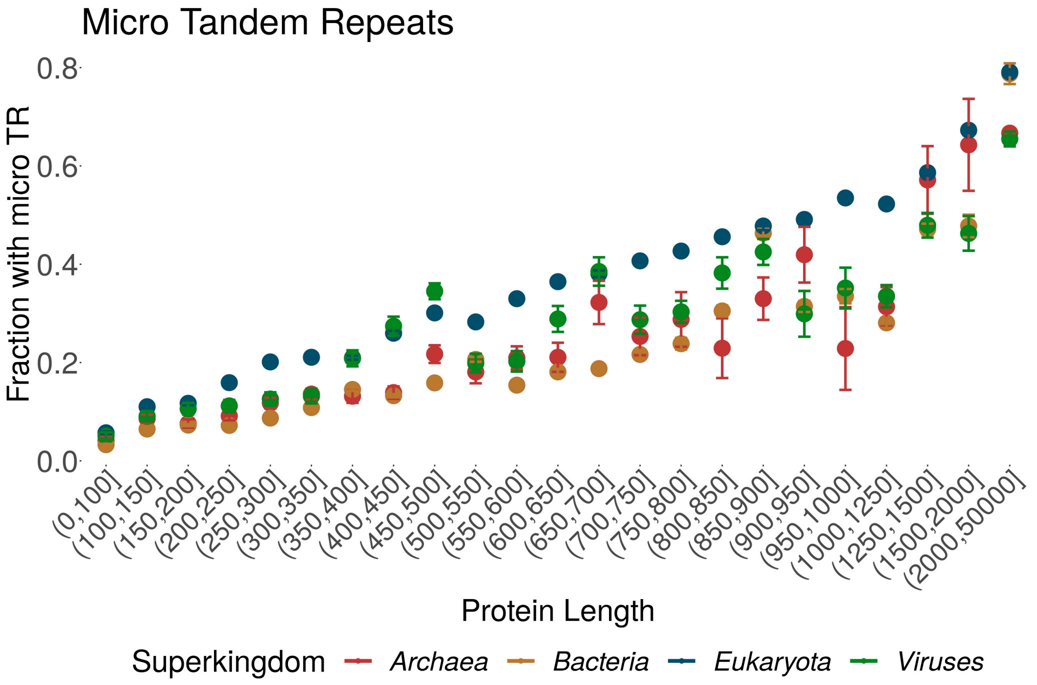

Figure S5. The fraction of proteins with micro TRs as a function of sequence length by kingdom resulting in a linear relationship.

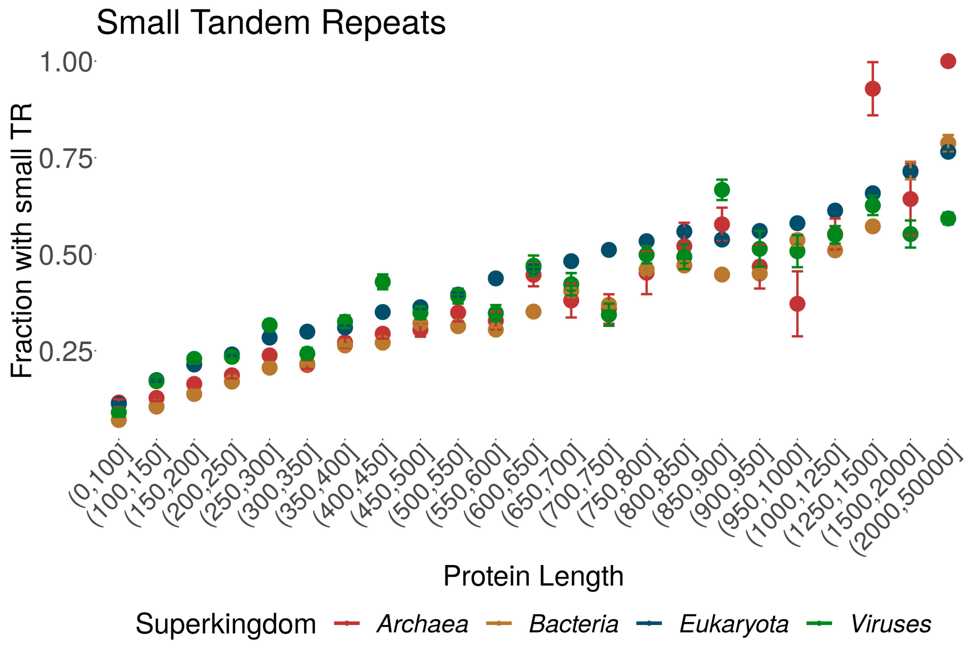

Figure S6. The fraction of proteins with small TRs as a function of sequence length by kingdom resulting in a linear relationship.

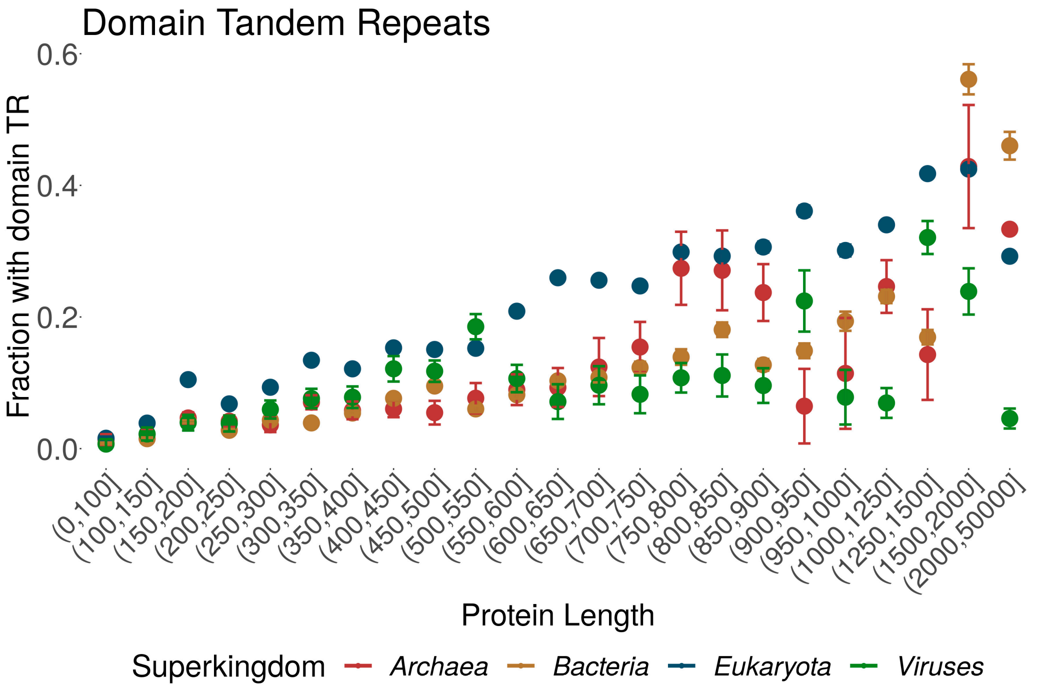

Figure S7. The fraction of proteins with domain TRs as a function of sequence length by kingdom resulting in a linear relationship.

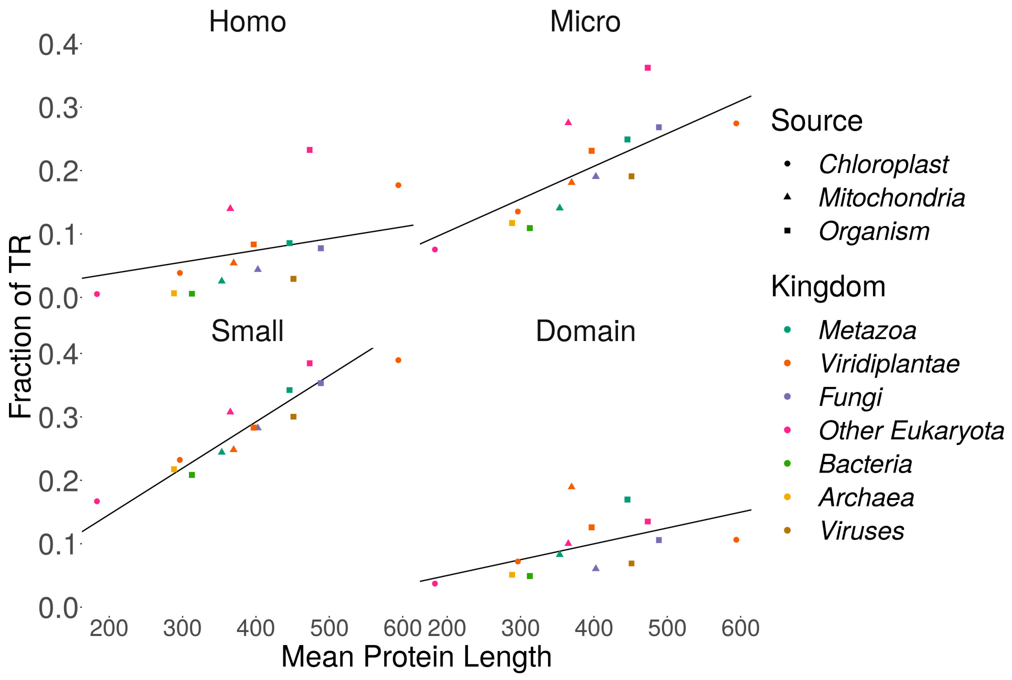

Figure S8. The amount of TRs (normalized by the amount of protein entries of the species) is displayed separately for each TR-type as a function of the mean length of the proteins. It can be clearly seen, that TRs appear mostly as small TRs. Comparing the fraction of TRs kingdom-wise, some clear tendencies can be seen for micro- and small TRs. For example, chloroplastic proteins with unknown Kingdom tend to have few TRs and short mean protein length. Where in contrast mitochondrial proteins from Viridi plantae and Fungi tend to have many TRs and long mean protein length.

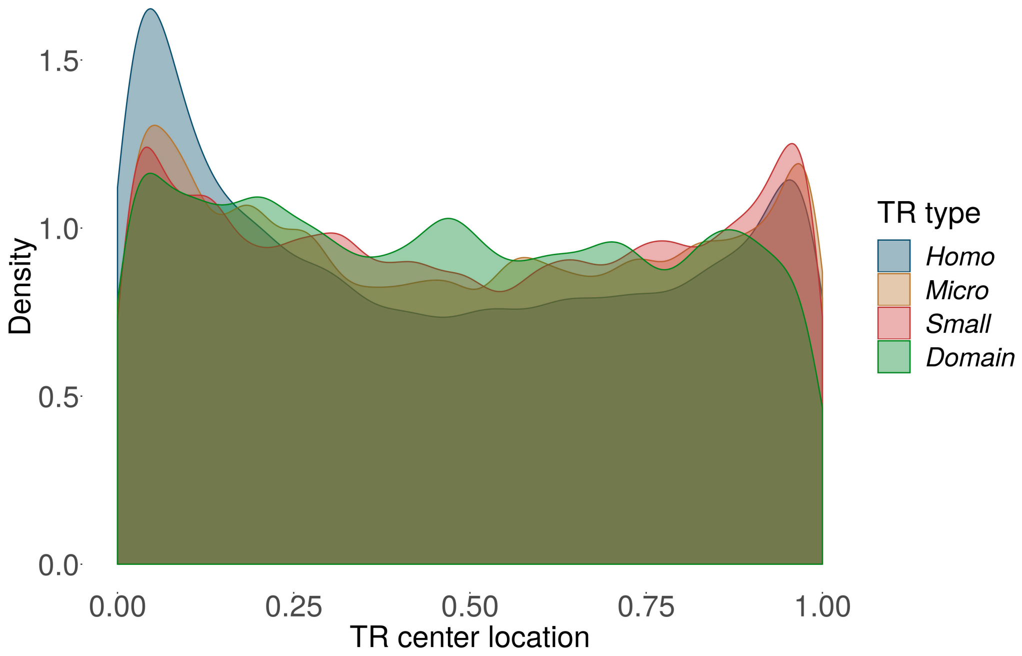

Figure S9. Density plot for the relative positions of TRs within proteins overall super kingdoms. The relative position refers with 0 to the N-terminus and with 1 to the C-terminus of a protein. Colours indicate repeat unit lengths. Interestingly, shorter TRs are biased towards the flanks of the protein.

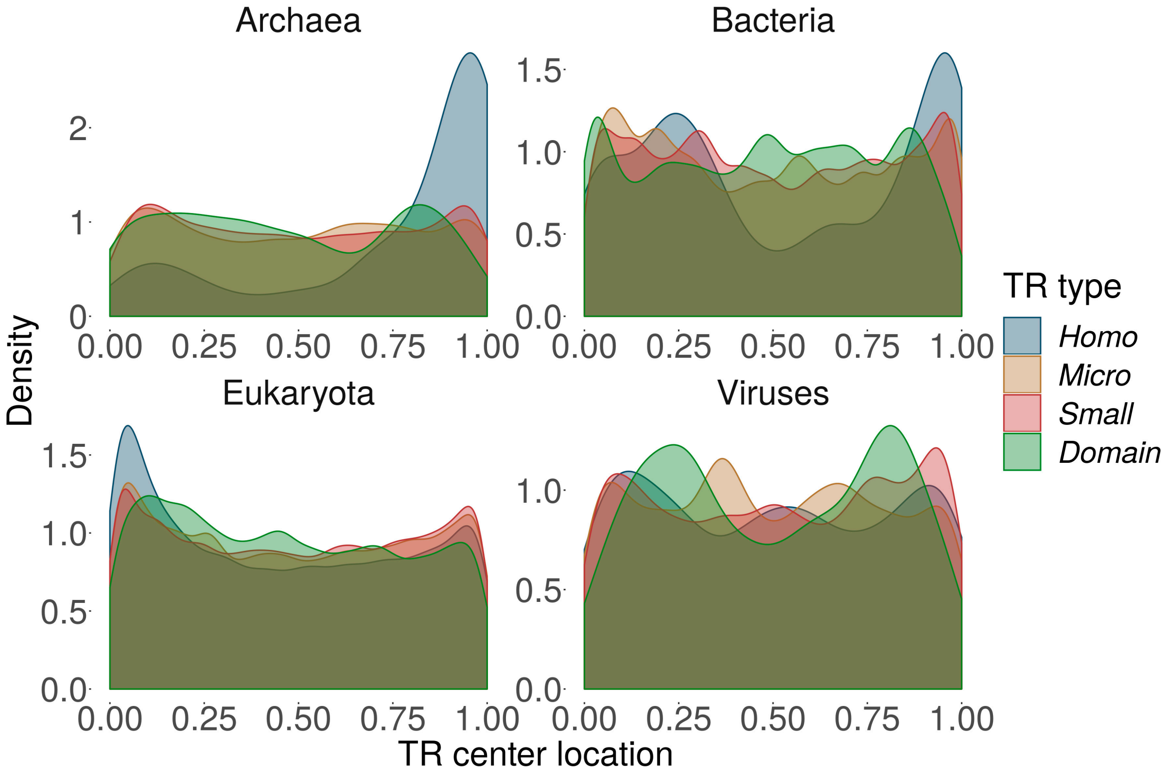

Figure S10. Density plots for the relative positions of TRs within proteins for four Superkindoms. The relative position referes with 0 to the N-terminus and with 1 to the C-terminus of a protein. Colours indicate repeat unit lengths. Interestingly, short TRs are biased towards the flanks of the protein. In particular for Eukaryotes, there is a clear correlation between TR unit length and location bias to the protein flanks. For Eukaryotes, TRs are particularly prevalent in the N-terminal protein flank. homorepeats in Archaea and, to a lesser degree, in Bacteria show a strong bias to the C-terminal protein flank.

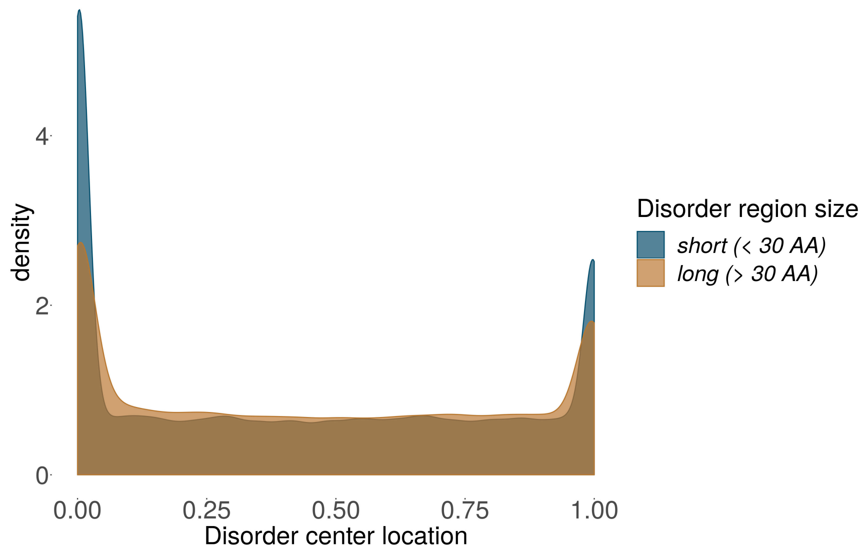

Figure S11. Density plots of position of disorder regions within the protein overall super kingdoms. Both short and long disorder regions tend to cluster towards the flank of proteins, to the N-terminal specifically.

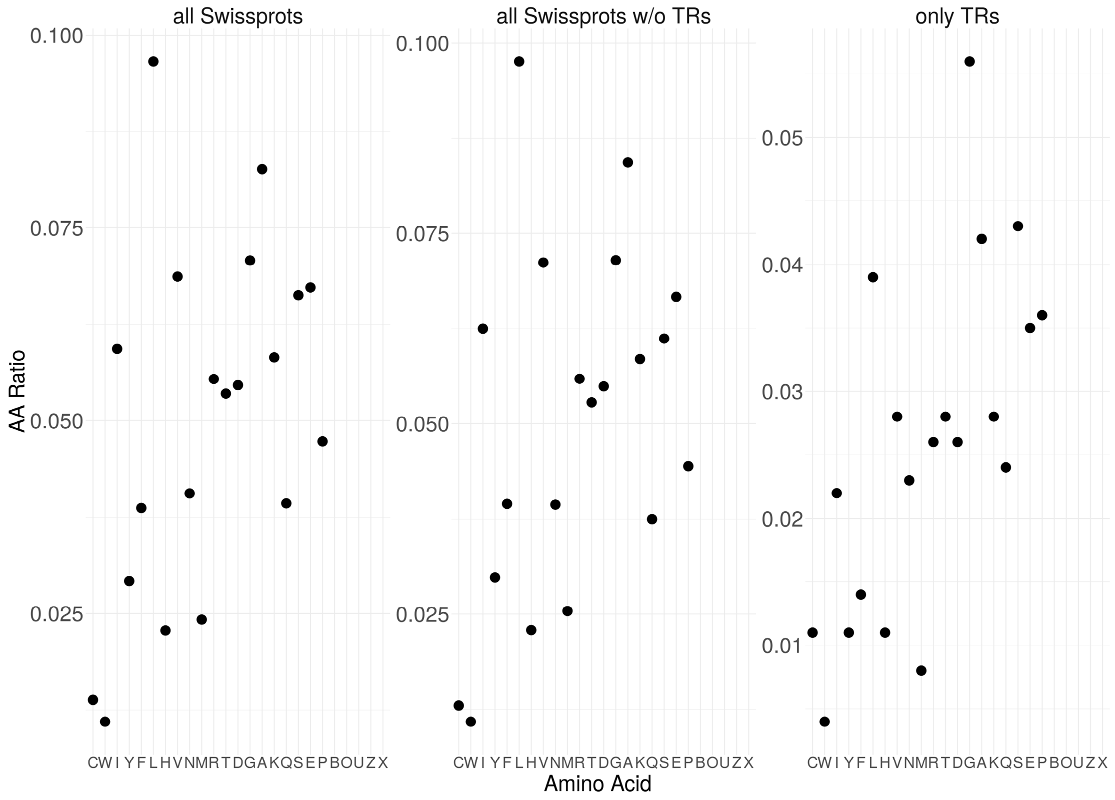

Figure S12. The amino acid ratio was calculated by the number of appearance of each amino acid divided by the overall number of amino acids per category and plotted against the amino acids in increasing disorder promoting potential. The group of all Swiss-Prots represents all protein sequences from Swiss-Prot. Of those, all proteins which have at least one detected TR were subtracted resulting in the group 'all Swissprots w/o TRs'. The group 'only TRs' was calculated by the multiple sequence alignment of the TRs. For the amino acids B, O, U, Z and X was no disorder potential available. One can see that the amino acid ratio of TRs shows a positive linear relationship with increasing disorder propensity. Disorder promoting residues seem to appear more often in TR sequences compared to overall protein sequences and to proteins without TRs.

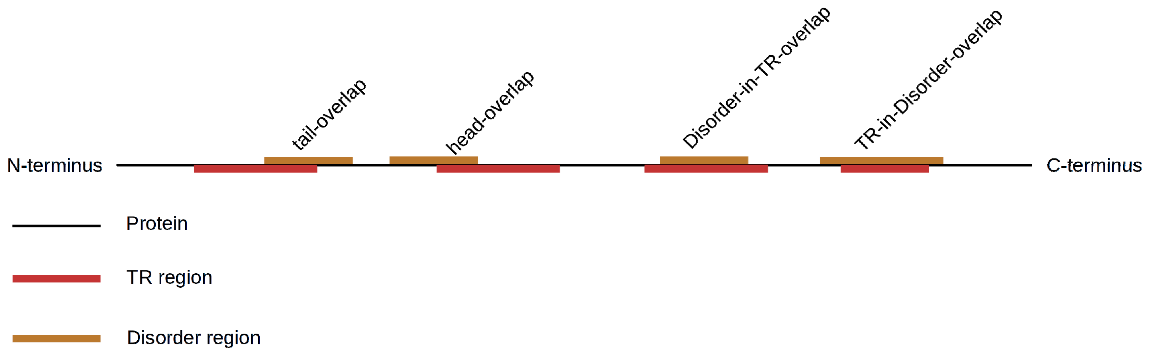

Figure S13. Overlap regions in proteins with intrinsic disorder and tandem repeats. We distinguish four different overlaps of IDR with TRs: *tail-overlap* where IDR begin within the TR-sequence and finishes after the TR-region. In contrast, we call *head-overlaps* overlap regions when the IDR begins before the TR-sequence and finishes within. If the IDR lies within a TR sequence, we call it. *Disorder-in-TR* and *TR-in-Disorder-overlap* if the TR-region lies within the IDR.

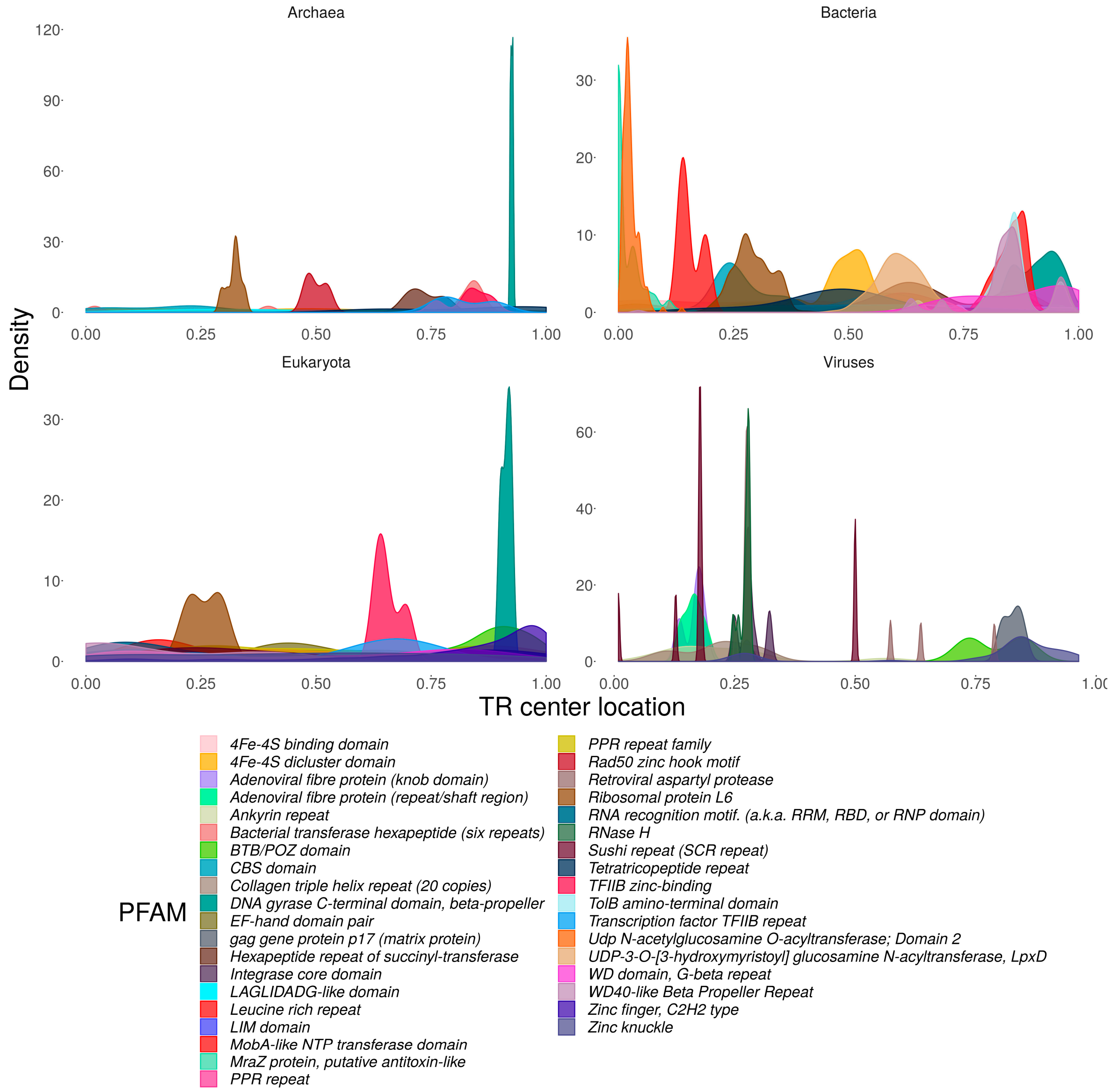

Figure S14. The ten PFAM with the most detected TRs for each superkingdom are plotted according their normalized TR center location (see Methods) and number of site-specific TRs. As expected, it can be seen that the protein family position is consistent overall superkingdoms.

Table S1. For each superkingdom are the ten most frequent PFAMs listed toghether with their PFAM Description and Accession number. 'Count' represents the number of appearances of the PFAM model in our data.

PFAM Name	PFAM Desc	PFAM Acc	count
**Archaea**			
TFIIB	Transcription factor TFIIB repeat	PF00382	35
CBS	CBS domain	PF00571	22
Fer4	4Fe-4S binding domain	PF00037	16
Fer4_7	4Fe-4S dicluster domain	PF12838	13
LAGLIDADG_3	LAGLIDADG-like domain	PF14528	11
Hexapep	Bacterial transferase hexapeptide (six repeats)	PF00132	9
TF_Zn_Ribbon	TFIIB zinc-binding	PF08271	9
Ribosomal_L6	Ribosomal protein L6	PF00347	7
Rad50_zn_hook	Rad50 zinc hook motif	PF04423	7
Fer4_10	4Fe-4S dicluster domain	PF13237	7
**Bacteria**			
Hexapep	Bacterial transferase hexapeptide (six repeats)	PF00132	928
MraZ	MraZ protein, putative antitoxin-like	PF02381	320
Ribosomal_L6	Ribosomal protein L6	PF00347	317
NTP_transf_3	MobA-like NTP transferase domain	PF12804	244
Hexapep_2	Hexapeptide repeat of succinyl-transferase	PF14602	223
PD40	WD40-like Beta Propeller Repeat	PF07676	164
Acetyltransf_11	Udp N-acetylglucosamine O-acyltransferase; Domain 2	PF13720	158
LpxD	UDP-3-O-[3-hydroxymyristoyl] glucosamine N-acyltransferase, LpxD	PF04613	127
TolB_N	TolB amino-terminal domain	PF04052	115
DNA_gyraseA_C	DNA gyrase C-terminal domain, beta-propeller	PF03989	100
**Eukaryota**			
WD40	WD domain, G-beta repeat	PF00400	1449
zf-C2H2	Zinc finger, C2H2 type	PF00096	828
LRR_8	Leucine rich repeat	PF13855	587
EF-hand_7	EF-hand domain pair	PF13499	520
RRM_1	RNA recognition motif. (a.k.a. RRM, RBD, or RNP do main)	PF00076	413
LIM	LIM domain	PF00412	260
PPR	PPR repeat	PF01535	226
PPR_2	PPR repeat family	PF13041	225
TPR_1	Tetratricopeptide repeat	PF00515	184
Collagen	Collagen triple helix repeat (20 copies)	PF01391	181
**Viruses**			
zf-CCHC	Zinc knuckle	PF00098	56
Gag_p17	gag gene protein p17 (matrix protein)	PF00540	37
RVP	Retroviral aspartyl protease	PF00077	13
Ank	Ankyrin repeat	PF00023	11
Adeno_knob	Adenoviral fibre protein (knob domain)	PF00541	11
Adeno_shaft	Adenoviral fibre protein (repeat/shaft region)	PF00608	11
rve	Integrase core domain	PF00665	11
BTB	BTB/POZ domain	PF00651	10
RNase_H	RNase H	PF00075	9
Sushi	Sushi repeat (SCR repeat)	PF00084	9

References

1. Marcotte, E.; Pellegrini, M.; Yeates, T.; Eisenberg, D. A census of protein repeats. *J. Mol.Biol.* **1999**, *293*, 151–160.

© 2020 by the authors. Submitted for possible open access publication under the terms and conditions of the Creative Commons Attribution (CC BY) license (http://creativecommons.org/licenses/by/4.0/).
